# Postpartum Chylothorax: A Case Report and Review of the Literature

**DOI:** 10.7759/cureus.47839

**Published:** 2023-10-27

**Authors:** Natalia Moguillansky, Ali Ataya

**Affiliations:** 1 Department of Medicine, Division of Pulmonary Critical Care, and Sleep Medicine, University of Florida Health, Gainesville, USA

**Keywords:** postpartum chylothorax, octreotide, thoracic duct, pleural effusion, post-partum chylothorax, chylothorax

## Abstract

Idiopathic postpartum chylothorax is an uncommon finding, with only four cases described in the literature. We present the case of a 37-year-old female who was diagnosed with chylothorax three days after the delivery of her baby. Chylothorax was managed with chest tube placement, a low-fat diet, and octreotide. As opposed to the current literature, her chylothorax resolved with medical management and chest tube insertion without further surgical intervention. The chest tube was removed 11 days after chest tube placement, and she was discharged in stable condition. We also review the most recent literature on postpartum chylothorax.

## Introduction

Chylothorax is characterized by the accumulation of chyle in the pleural space and is caused by a disruption of the thoracic duct. Classically, it is classified as traumatic vs. non-traumatic [1]. Chylothorax is an infrequent complication after childbirth. It has been hypothesized that the Valsalva maneuver during labor may injure the thoracic duct, causing the chylothorax [2]. There are four prior reported cases of postpartum chylothorax that were treated with different modalities [2-5]. We present the first case of postpartum chylothorax in the literature that resolved only with medical management and chest tube insertion.

## Case presentation

A 37-year-old female, gravida 2, para 1 (G2P001), presented to the labor and delivery unit at 35 weeks and four days of gestation with signs and symptoms of preeclampsia with severe features, based on blood pressure and thrombocytopenia. She had a history of open-angle glaucoma, major depressive disorder, congenital factor VII deficiency, alcoholism, and cigarette smoking, and her urinary drug screen was positive for cannabinoids. She initially denied dyspnea, headaches, visual changes, or epigastric pain.

On admission, her blood pressure was 154/88 mmHg, her pulse was 88 beats per minute, and her oxygen saturation was 98%. Her pulmonary, abdominal, cardiovascular, and neurological exams were normal.

**Figure 1:**
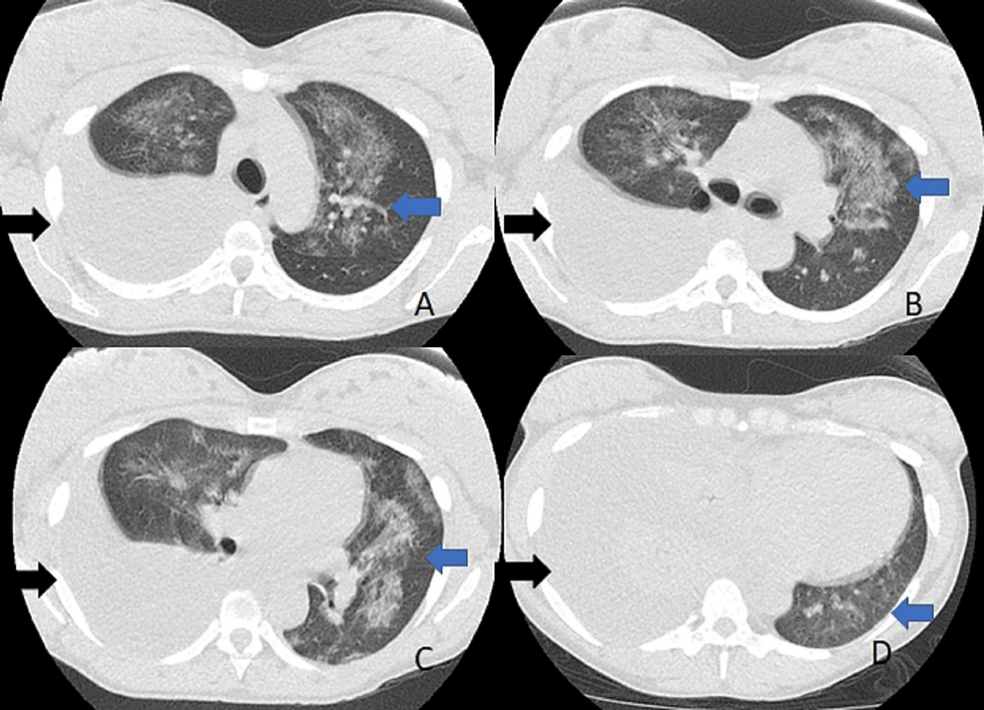
Initial CT of the chest and lung windows Right pleural effusion (black arrows) and pulmonary infiltrates (blue arrows). A. upper lobes; B. carina; C. middle lobe; D. lower lobes

**Figure 2:**
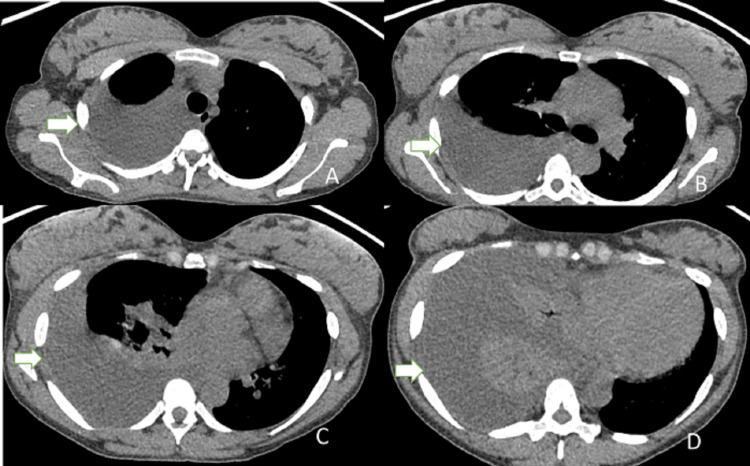
Initial CT of the chest and mediastinal windows Right pleural effusion (white arrows) A. upper lobes; B. carina; C. middle lobe; D. lower lobes

She was started on a magnesium infusion, which was maintained for 36 hours. Two days after admission, she was induced due to hypertension and had a spontaneous vaginal delivery. Two days after delivery, she started complaining of shortness of breath and chest pain and had oxygen requirements of 2 liters per minute. She had decreased breath sounds on the right lower thorax. The chest X-ray showed left parahilar patchy airspace opacities and a large right pleural effusion with underlying airspace opacities. Subsequent computed tomography (CT) of the chest showed a large right pleural effusion with compressive atelectasis of the right lung and patchy airspace disease in both upper lobes (Figures 1-2).

She was started on antibiotics for a positive *Haemophilus influenzae* based on a sputum pneumonia polymerase chain reaction (PCR) test. Three days after delivery, a thoracentesis was performed, showing a milky white fluid. For this reason, the procedure was converted to thoracostomy tube placement, with an initial output of 1.3 liters. Pleural fluid analysis showed a white blood cell count of 300 CUMM, a red blood cell count of 810 CUMM, neutrophils of 79%, lymphocytes of 5%, macrophages of 16%, protein <1.5 g/dl, lactate dehydrogenase (LDH) of 75 U/L, pH of 7.56, glucose of 100 mg/dl, and triglycerides of 474 mg/dl, consistent with transudate chylothorax. After thoracentesis, her oxygen requirements actually increased, and a repeat CT chest showed multifocal ground glass opacities and septal thickening, consistent with pulmonary edema. The pulmonary edema was thought to be secondary to preeclampsia, as the infiltrates were present prior to the thoracentesis, and the echocardiogram showed normal left ventricular function. She was transferred to the ICU, where she required intubation. A repeat bronchoalveolar lavage was performed, which was negative for bacterial, fungal, and acid-fast bacilli (AFB) cultures with negative pneumonia PCR and cytology. She was diuresed and eventually extubated.

**Figure 3:**
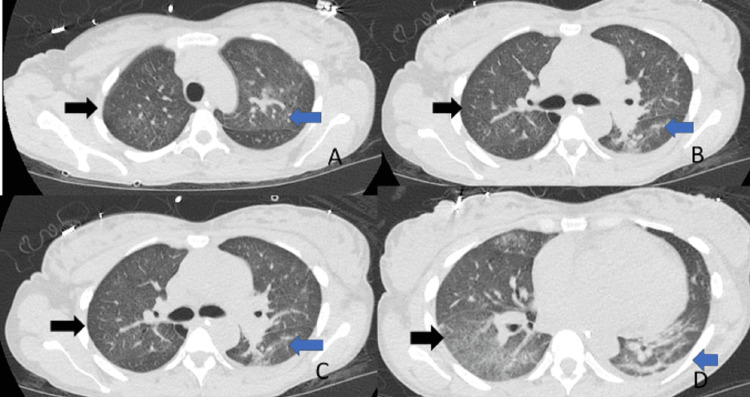
A follow-up CT of the chest after eight days of chest tube placement Lung windows, resolution of right pleural effusion (black arrows), and residual pulmonary infiltrates (blue arrows). A. upper lobes; B. carina; C. middle lobe; D. lower lobes

**Figure 4:**
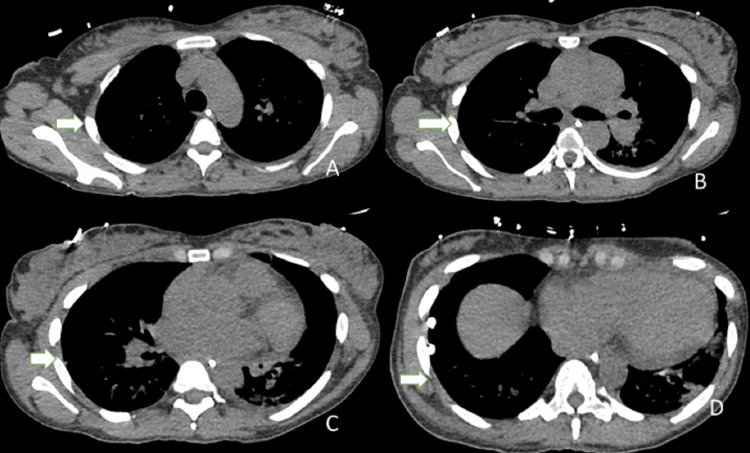
A follow-up CT of the chest after eight days of chest tube placement Mediastinal windows and resolution of right pleural effusion (white arrows). A. upper lobes; B. carina; C. middle lobe; D. lower lobes

For the treatment of the chylothorax, she was started on octreotide 50mg subcutaneously every eight hours and a low-fat diet. On the first day, the chest tube output was 1540 ml. Subsequently, the chest output decreased to less than 300 ml/24 hours until day 11, when the chest tube was removed. An interventional radiology service was consulted for a possible lymphangiogram. However, due to prolonged international normalized ratio (INR) and prothrombin time due to congenital factor VII deficiency (GU1), the procedure was not done. A magnetic resonance angiography (MRA) of the chest with IV contrast showed resolution of the pleural effusion and pulmonary infiltrates without evidence of lymphatic malformation in the chest. A CT of the chest eight days after chest tube placement showed improvement in the right pleural effusion and pulmonary infiltrates (Figures 3, 4).

Due to low chest tube output, nine days after chest tube placement, she was re-started on a regular diet to challenge the chylothorax. Her chest tube output remained low, and the chest tube was removed after 11 days of chest tube placement. Of note, had her chest tube output increased, she would have required further surgical intervention, in spite of her coagulopathy. She was discharged home in stable condition 20 days after admission.

## Discussion

Causes of chylothorax can be divided into traumatic and non-traumatic. Traumatic causes include iatrogenic procedures such as esophagectomy and pulmonary resection, penetrating trauma, and blunt thoracic trauma [1, 6]. Common non-traumatic causes include malignancy, especially lymphoma. Chylothorax can present with shortness of breath, coughing, and chest discomfort. Pleural fluid is described as having a “milky” appearance. Triglyceride levels of more than 110 mg/dl in the pleural fluid are diagnostic of chylothorax. Management can be divided into conservative, medical, surgical, or percutaneous lymphatic interventions. Recent recommendations by Agrawal et al. suggest conservative management if the pleural fluid output is less than 500 ml/24 and surgical or interventional management if it is more. Conservative management includes a low-fat diet and somatostatin, or its analogs such as octreotide. Surgical interventions consist of controlling the chyle leak via ligation of the thoracic duct and controlling the pleural effusion via thoracentesis, chest tube placement, and pleurodesis. Percutaneous lymphatic interventions involve performing lymphangiography to identify the site of injury or leak and, finally, thoracic duct embolization across the site of leak [1].

It has been proposed that disruption of the thoracic duct during delivery occurs due to the high intrathoracic pressures generated by the Valsalva maneuver during delivery [2].

The first case of postpartum chylothorax was published in 1987 by Tornling et al. [3]. They described the case of a 23-year-old female who developed dyspnea two weeks after delivery. Twenty-three days after delivery, she was diagnosed with left-sided chylothorax. A chest tube was placed, and she was treated with a fat-deficient diet supplemented with medium-chain triglycerides for three weeks. Because the chest tube output did not diminish, she required two surgical thoracotomies to successfully treat her chylothorax. The second case was published in 1991 by Cammarata et al. [2]. They reported a 20-year-old who developed a right pleural effusion post-delivery. Thoracentesis three weeks later showed chylothorax. She had a chest tube placed and was treated with a low-fat diet. She had a persistent chest tube output of 1 L/day, so she was taken for thoracotomy and thoracic duct ligation. She also had tetracycline pleurodesis. Although she initially improved, she had persistent loculated effusions two years after follow-up. The third case was reported in 2008 by Momose et al. [4]. They describe the case of a 24-year-old female who developed a right chylothorax diagnosed by thoracentesis two months after childbirth. They performed chest lymphoscintigraphy, which showed abnormal tracer accumulation in the right hemithorax. Subsequently, they performed a single photon emission computed tomography/computed tomography (SPECT/CT), which showed the abnormal tracer reflux of lymph fluid in the right dorsal pleural wall. She underwent video-assisted thoracoscopic surgery (VATS), which confirmed she had rips in the lymph ducts near their entry site into the right hemothorax. The ducts were ligated. The last case was published in 2022 by Streit et al. [5]. They reported a case of a 31-year-old female who presented with left thoracic pain one month following childbirth. The diagnosis of left chylothorax was made by thoracentesis. An MRI without contrast showed normal anatomy of the thoracic duct. A lymphoscintigraphy revealed a chyle leak at the T10 level. Three months after a long-chain triglyceride-free diet, due to a lack of improvement, she underwent surgical thoracic duct ligation. A recurrence of the chylothorax occurred after the resumption of a regular diet. For this reason, she underwent a lymphography, which identified the leak of contrast. This examination allowed us to guide the location of the thoracic duct embolization. Follow-up at seven days did not show evidence of chylothorax relapse.

As noted above, the four cases published in the literature required surgical and percutaneous lymphatic interventions. This may be a publication bias, as less complex cases tend not to be published. Our case is unique in that the chylothorax resolved with medical management and chest tube insertion without any further, more invasive surgical intervention.

## Conclusions

Postpartum chylothorax is a rare cause of pleural effusion. Management of chylothorax can be divided into conservative or medical, surgical (thoracic duct ligation), or percutaneous lymphatic interventions (lymphangiography and thoracic duct embolization). Four cases of postpartum chylothorax were published in the literature. All of them required surgical intervention, and one required percutaneous lymphatic treatment. We present the fifth case of postpartum chylothorax in the literature and the first of its kind, which resolves with medical management and chest tube insertion without any further, more invasive surgical intervention.
